# Spatial Distribution and Pollution Assessment of Metals in Sediments of the Babon River, Central Java, Indonesia

**DOI:** 10.1155/2024/2065513

**Published:** 2024-07-29

**Authors:** Haeruddin Haeruddin, Agoes Soegianto, Frida Purwanti, Arif Rahman, Carolyn Melissa Payus, Hefni Effendi

**Affiliations:** ^1^ Universitas Diponegoro Departemen Sumber Daya Akuatik, Semarang, Central Java, Indonesia; ^2^ Universitas Airlangga Departemen Biologi, Surabaya, East Java, Indonesia; ^3^ Universiti Malaysia Sabah Fakulti Sains dan Sumber Alam, Kota Kinabalu, Sabah, Malaysia; ^4^ Institut Pertanian Bogor (IPB University) Fakultas Perikanan dan Ilmu Kelautan, Bogor, West Java, Indonesia

## Abstract

**Background:**

The Babon River is one of the drainage channels in Semarang, Indonesia, that is used for drinking water, irrigated agriculture of paddy fields and gardens, fish farming media, and the disposal of industrial wastewater. This study aimed to assess the spatial distribution of metals in the sediment of the Babon River through the utilization of different pollution indices. These indices included concentration factor (Cf), contamination factor (CF), enrichment factor (EF), geoaccumulation index (Igeo), sediment pollution index (SPI), threshold effect level (TEL), and probable effect level (PEL).

**Methods:**

Seven sampling locations were sampled for water and sediment in April, June, and September of 2021. The measurement of the metal was conducted using an atomic absorption spectrophotometer.

**Results:**

The findings of the study demonstrated that sediments with a predominant sand texture exhibited higher levels of Cd and Pb, while the sediments characterized by a predominant clay texture had a higher concentration of Cr. Upon analyzing the Cf, CF, EF, Igeo, and SPI data, it has been confirmed that the sediment in the Babon River has substantial levels of Cd, Cr, and Pb. According to the TEL and PEL values, it can be observed that the presence of Cd and Pb has detrimental effects on the biological health of the benthic biota.

**Conclusions:**

The aquatic biota and the benthic environment may be subject to adverse effects in the event where the concentration of metals in the sediment is higher than the natural concentration of metals.

## 1. Introduction

The river is one of the water resources that is most vulnerable to the effects of human activity, which might result in the degradation of the aquatic ecosystem [1]. The Babon River is an important surface water body that traverses Semarang city (the capital of Central Java), Indonesia. This watercourse is an important resource for the surrounding communities. The river water serves several purposes, including its utilization as a primary supply of drinking water, for the irrigation of paddy fields and farms, as a supply of water for fish farming, and for industrial purposes. The river is also used as a drainage channel and as a location to dispose of wastewater from community activities in the river's vicinity; all of these activities have the potential to pollute the river with metals. In sediments and water bodies that have been polluted, metals such as Cd, Cr, Pb, and Fe have been discovered [2]. The presence of metals has been shown to have a negative impact on plankton [3], nekton [4, 5], and benthos [6–8]. People who consume water, animal meat, or aquatic plants containing these metals may develop health issues [9, 10], which can potentially lead to an elevated risk of developing cancer over their lifetime [11].

Due to their high toxic ability, availability, and persistence, metals have become a worldwide issue in the aquatic ecosystem [12, 13]. These contaminants accumulate in the aquatic environment and are then incorporated in high concentrations into the sediments of the aquatic environment [14]. The concentration of metals in sediments is typically three to five times greater than in water [15]. Consequently, sediment analysis can be used to identify metals more quickly than water analysis, which can be used to quantify metal concentration [16]. As a result, sediments are regarded as a potential indicator for evaluating the condition of aquatic environments [17]. Locations with rapid sediment deposition close to a contaminated source have the highest metal concentrations in the sediment [18]. Mineral content, sediment texture, physical mobility, redox state, the amount of organic matter present, and biological activity are some of the elements that have the potential to influence the dispersion of pollutants in sediment [19]. Furthermore, sediments serve as a record of human causes of pollution such as shipbuilding industries [20].

The objective of this study was to examine the spatial distribution of Cd, Cr, Pb, and Fe in the Babon River sediments, categorize their concentration and contamination factors, evaluate their source using enrichment factors, assess their risk to benthic organisms using threshold effect level (TEL) and probable effect level (PEL), and determine and compare the sediment contamination status using geological indices (Igeo) and sediment pollution index (SPI). SPI was developed as a method for assessing sediment pollution by combining data on pollutant concentrations and their potential impacts on benthic organisms [21, 22].

## 2. Materials and Methods

### 2.1. Sampling of River Water and Sediment

**Figure 1:**
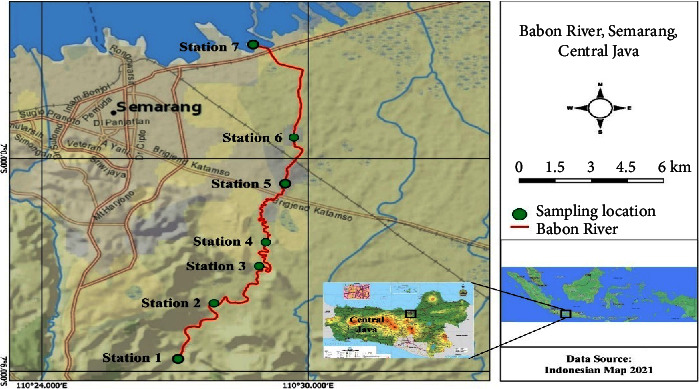
Locations of sampling stations for sediment and water samples in the Babon River, Central Java, Indonesia.

A total of seven sampling locations (stations) were set up to collect sediment and water samples from the Babon River. Site 7 is situated in the upper section of the river, while site 1 is located downstream (Figure 1). The remaining stations are situated between the river's upstream and downstream sections. The geographic coordinates for each sampling site are as follows: station 1 (7.094209 S; 110.45126 E), station 2 (7.070023 S; 110.4633 E), station 3 (7.053744 S; 110.48076 E), station 4 (7.039502 S; 110.48425 E), station 5 (7.011687 S; 110.4914 E), station 6 (6.989124 S; 110.49446 E), and station 7 (6.946375 S; 110.47969 E). The choice of sampling sites was based on an assessment of probable contamination levels and the degree of human activity in the vicinity of the sites (Table 1). At each sampling site, three adjacent sampling points were selected to collect water and sediment samples. Three separate samplings were conducted at each sampling site for both water and sediment. These samplings took place in April, June, and September of 2021. As a result, each sampling site in our study consists of nine water and sediment samples.

Using Kemmerer bottles, river water samples were collected and then transferred to clean 1-liter PE sample bottles, two bottles per sampling location. Water samples intended for metal determination were passed via filter paper (with a pore size of 0.45 *µ*m) immediately after collection in the field. The filtering apparatus is prerinsed with 5% suprapure HNO_3_ (Suprapur, Merck, Darmstadt, Germany), and then washed with approximately 300 ml of double deionized water prior to filtering of each individual sample. For metal analysis, suprapure HNO_3_ was used to acidify the water to a pH of less than 2. A cold box that was maintained at a temperature of 4°C was then used to transfer river water samples to the laboratory.

A Van Veen grab was used in order to collect samples of sediment. In each location, three contiguous sampling points were chosen for sediment samples. These samples were then combined in a plastic pan and mixed thoroughly using a plastic spoon. A subsample of approximately 300 grams is retained in a 500 ml wide-mouth high-density polyethylene bag for metal analyses. Two bags of sediment were collected per sampling site. Then, samples of the sediment were shipped to the research facilities in a cold box that was maintained at a temperature of 4°C for further examination.

### 2.2. Metal Analysis in Water and Sediment Sample

The analytical procedure for metals in seawater follows the guidelines outlined by USEPA [2] and Yüksel et al. [23–25]. An atomic absorption spectrophotometer (AAS) (Shimadzu, AA-7000, Tokyo, Japan) was used in order to determine the levels of the metals (Cd, Cr, Pb, and Fe) that were present in the filtered water sample. The units of measurement were mg/L. The metal detection limits for Cr, Cd, Pb, and Fe were 0.005 mg/L, 0.001 mg/L, 0.008 mg/L, and 0.05 mg/L, respectively. With the use of the seawater-certified reference material (NASS-7), which was supplied by the National Research Council of Canada (NRCC), the analytical performance was assessed in terms of its precision as well as accuracy. Analysis of NASS-7 produced the metal recoveries that are presented in Table 2.

The analytical methodology used in the determination of metals in sediment follows the standards provided by USEPA [26] and Yüksel et al. [23–25]. Samples of sediment were permitted to dry at ambient temperature before being sieved via a 2 mm mesh. A grinder was then used to pulverize the sediment samples. One gram of the crushed sediment sample was carefully placed into a digesting jar. The sample was then digested using a mixture of HCl and H_2_NO_3_ (Suprapur, Merck, Darmstadt, Germany) in a specific ratio (1 : 3). This digestion process took place in a microwave digester (MARS 6, CEM Corporation, USA) for a duration of four hours, maintaining a temperature of 90°C. Following the sample's cooling to the ambient temperature, it underwent filtration via filter paper (Whatman, with a pore size of 0.45 *μ*m). To achieve a final volume of 50 mL, deionized water was added. The AAS was used to analyze the concentrations of Cd, Cr, Pb, and Fe. Two separate measurements were taken of each sample, and then an average value was determined. The units of measurement were mg/kg. The metal detection limits were as follows: 0.01 mg/kg for Cr, 0.02 mg/kg for Cd, 0.01 mg/kg for Pb, and 0.03 mg/kg for Fe.

An analytical blank was analyzed using the same procedure that was used to analyze the sample. The standard solutions were similarly formulated using the acid matrix that was used to determine the sample concentrations. The precision and accuracy of the analytical performance were evaluated by measuring the certified sediment reference material (PACS-3), which was provided by the National Research Council of Canada (NRCC). The analysis of this reference material was conducted using the identical techniques that were used to analyze the samples. Analysis of PACS-3 produced the metal recoveries that are presented in Table 3. All of the metal standard solutions (Certipur, Merck, Darmstadt, Germany) possessed a high standard of analytical quality.

### 2.3. Analyzing Sediment Samples to Estimate Sand, Silt, and Clay Proportions

The sediment is initially filtered using a 2 mm screen (no. 10) and then filtered using a 75 *µ*m screen (no. 200) in order to determine the proportions of sand, silt, and clay. The pipette method was subsequently applied to the sediment samples that had passed through screen no. 200 in order to ascertain the ratios of silt and clay [27, 28].

### 2.4. Data Analysis

SPSS Statistics 25 (IBM Corporation, USA) was used to conduct the statistical analysis. The Shapiro–Wilk test demonstrated that the data on the concentration of metals in both river water and sediment did not exhibit a normal distribution. To evaluate the differences between the various metals present in sediment from multiple sampling locations, a Kruskal–Wallis test was employed, with a significance level of *P* < 0.05. A Spearman correlation evaluation was carried out in order to ascertain the degree of significant correlations that exist between the concentration of metals and the textures of the sediment.

The equation utilized to calculate the concentration factor (*C*_*f*_) of metals in sediment is expressed as follows:

(1) $$\begin{array}{c} C_{f}=\frac{C_{s}}{C_{w}}, \end{array}$$

where *C*_*f*_ is the concentration factor, *C*_*s*_ is the metal concentration in sediment samples (mg/kg), and *C*_*w*_ is the metal concentration in water samples (mg/l).

The formula used to establish the contamination factor (CFi) is based on Hakanson's [29] method and can be expressed as follows:

(2) $$\begin{array}{c} \text{CF}i=\frac{C_{i}}{B_{i}}, \end{array}$$

where CFi represents the contamination factor metal-i, *C*_*i*_ represents the concentration of metal-i in sediment samples (mg/kg), and *B*_*i*_ represents the concentration of metal-i in uncontaminated sediment samples (mg/kg).

The CF could be categorized as follows: unpolluted (CF < 1), moderately polluted (1 ≤ CF < 3), heavily polluted (3 < CF < 6), and highly polluted (CF > 6) [30, 31].

The sediment's enrichment factor (EF) was calculated by comparing each element's concentration to a control sample. This analysis aimed to identify potential sources of the elements, such as anthropogenic, crustal, geogenic, or lithologic e.g., manganese (Mn), aluminum (Al), and iron (Fe) in a specific sample equation (3) When the enrichment factor exceeds one, it suggests that the element of interest is derived from additional human-related sources. The current research used Fe as the normalizing factor [32]. Qingjie et al. [33] and Savosko et al. [34] identified five contamination categories that exhibited an association with the enrichment factor: unpolluted (EF < 2), slightly polluted (2 < EF < 5), moderately polluted (5 < EF < 20), heavily polluted (20 < EF < 40), and extremely polluted (EF > 40). The calculation formula is as follows:

(3) $$\begin{array}{c} \text{EF}=\frac{\left(\text{metal}/\text{Fe}\right)\text{ sediment}}{\left(\text{metal}/\text{Fe}\right)\text{ crust}}. \end{array}$$

Muller [35] established a methodology known as the geoaccumulation index (Igeo) to assess metal pollution by comparing present concentrations with preindustrial levels. The formula for Igeo, as described in equation (4), classifies the values of Igeo into six distinct pollution degree groups. The following are the six categories of pollution degrees: unpolluted (Igeo < 1), slightly polluted (1 < Igeo < 2), moderately polluted (2 < Igeo < 3), moderately-heavily polluted (3 < Igeo < 4), heavily polluted (4 < Igeo < 5), and extremely polluted (Igeo > 5).

(4) $$\begin{array}{c} \text{Igeo}=\frac{\log\;2\left(M\right)i}{1.5\left(M\right)r}, \end{array}$$

where (*M*)*i* represents the metal concentration found in sediment samples, whereas (*M*)*r* represents the average crustal metal concentration. Taylor [32] reported the following background concentrations: Cd = 0.15 mg/kg, Cr = 102 mg/kg, Pb = 14 mg/kg, and Fe = 56300 mg/kg. The analysis incorporates a factor of 1.5 to account for minimal human impacts and potential fluctuations in the background levels of certain metals in the environment.

The Igeo is an approach used to assess the presence of metal pollution in sediments by emphasizing a single metal. An alternative methodology known as sediment pollution index (SPI) has been proposed to comprehensively evaluate the state of sediment by considering the total number of metals, while also taking into account the weight of each metal toxicity (equation (5)). Metal toxicity weights are inversely related to average shale lithogenic limits and based on metal toxicity. Unpolluted sediments should not exceed typical shale metal concentrations. The elements with lower levels of hazard, such as Cr, were assigned a weight of 1. The estimated toxicity weights for Pb and Cd were 5 and 300. The SPI can be expressed as the following equation [22]:

(5) $$\begin{array}{c} \text{SPI}=\frac{\sum \left(\text{CFi}*\text{Wi}\right)}{\sum \mathrm{Wi}}. \end{array}$$

The variables CFi and Wi represent the contamination factor and toxicity weight of metal *i*, respectively. The sediment pollution index (SPI) is a category consisting of five classes that assess the quality of sediments, ranging from natural to dangerous levels. According to the estimation provided, the classifications are as follows: natural sediments (SPI = 0–2), low contaminated sediments (SPI = 2–5), moderately polluted sediments (SPI = 5–10), highly polluted sediments (SPI = 10–20), and hazardous sediments (SPI > 20).

A rating system named the Sediment Quality Rating (SQR) was established by the National Oceanic and Atmospheric Administration [36] with an objective to evaluate the quality of sediment in relation to metal pollution. In considering the probable effect level (PEL) and the threshold effect level (TEL) values, the level of metal pollution is taken into account to determine this rating. Metal concentrations that are lower than the TEL are assigned a rating of 1, while those that fall between the TEL and PEL have ratings of 2–4. Those beyond the PEL are assigned a rating of 5 (Table 4).

## 3. Results

### 3.1. Metal Distribution and Concentration in River Water and Sediment

The concentrations of metals (Cd, Cr, Pb, and Fe) in river water and sediment at different sampling locations are presented in Table 5. The Kruskal–Wallis test provides insignificant differences in the concentrations of Cd, Cr, and Pb in river water and sediment across different locations. Likewise, there were no significant differences in the concentrations of Fe measured in the river water across the sampling locations. Nevertheless, there was a significant difference in Fe concentrations in river sediment among the sampling locations (Table 5).

### 3.2. Correlation between Sediment Texture and Metal Concentration

**Figure 2:**
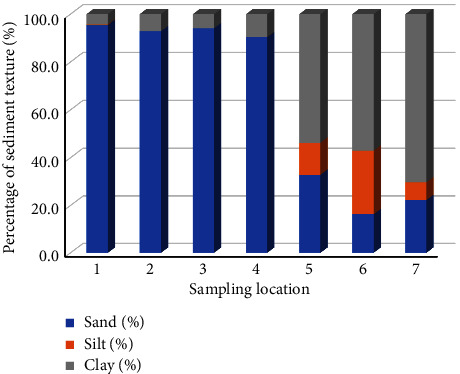
Percentage of the sediment texture at each sampling station.

The composition of sediment texture in the Babon River demonstrates substantial variation across its upstream and downstream sections (Figure 2). The analysis of sediment texture, carried out from sampling location 1 in the upstream region to sampling location 4 in the middle region, exhibited an elevated percentage of sand, with the percentage ranging from 84.4% to 98.4%. In contrast, the analysis of sediment texture at sampling locations 5–7 in the downstream region indicated a prevalent clay composition that varied between 4.4% and 84% (Figure 2).

The correlation between the concentration of metals in sediments and the sediment texture is shown in Table 6. Elevated concentrations of Cd and Pb were observed in sediments characterized by a dominant sand texture, which was confirmed by correlation values of 0.512 and 0.388, respectively. The sediments characterized by a predominant clay texture have a higher concentration of Cr confirmed by a correlation value of 0.263. The presence of sediments predominantly composed of sand texture has a positive correlation value of 0.478 with elevated concentrations of Fe.

### 3.3. Sediment Quality and Sediment Pollution Index

The metal concentration factor (Cf) of the sediment provides a reflection of the proportion of metal concentration in the water that becomes concentrated in the sediment.  Table 7 demonstrates that sampling site 1 has the greatest concentration factor of Cd and Fe compared to the other sampling sites. Sampling site 2 exhibits the highest concentration factor of Cr and Pb in comparison to the other sampling sites.

The metal CF of the sediment is defined as the proportion of metal concentration in the observed sediment to that in naturally occurring sediments. The CF of various metals observed in sediments across various sampling locations is represented in Table 8. Almost all sampling locations are highly polluted by Cd and Pb (Table 8). Sampling sites 4, 5, 6, and 7 are moderately polluted by Cr.

Human activities' effects on sediment's metal content were assessed using the enrichment factor (EF).  Table 9 shows how EF values can identify sediment metal contamination sources. The enrichment factor (EF) for the different metal types present in sediments, with the exception of Fe, exceeds a value of 40, which means that it is extremely polluted (Table 9).

The Igeo values of several metal types in the sediments taken from various locations are presented in Table 10. The average values of Igeo suggest that sediments collected from all sampling sites in the Babon River were typically unpolluted by Cr and Fe. In addition, sampling sites 1, 2, and 6 were found to be unpolluted from Pb. Sampling sites 1–7 were categorized to exhibit a moderate level of Cd pollution, whereas sample sites 3, 4, 5, and 7 were classed as having a modest level of Pb pollution.

**Figure 3:**
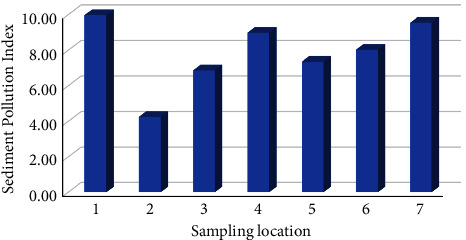
Sediment pollution index (SPI) of multiple metals at the sampling locations.

The SPI class variation of the Babon River sediment is illustrated in Figure 3. The examination indicates that the sediments of the Babon River are classified into two categories: moderately (sampling site 1 and 3–7) or low polluted (sampling site 2).

## 4. Discussion

### 4.1. Sediment Pollution Assessment

The observed levels of metal concentrations in river water have been found to be comparatively lower than those observed in river sediment. As per Rosado et al. [37], sediment acts as a primary sorbent pollutant, which contains pollutant concentrations that could surpass those present in water. Sediment plays an essential role in absorbing pollutants that enter the environment, particularly trace metals, and can accumulate in significant amounts in the sediment of aquatic ecosystems, which are the habitat of various benthic organisms [38, 39].

Fe is a metallic element that shows the highest levels of Fe present in the water and sediments of the Babon River, followed by Pb, Cr, and Cd. Given that Fe is an element that is abundant in the crustal layer, the elevated levels of Fe in the Babon River are apparent. However, compared to the background level [32, 40, 41], Babon River sediment has lower Fe and Cr concentrations and higher Cd and Pb concentrations. It is apparent that the elevated concentrations of Cd and Pb in the Babon River can be due to both natural and human-caused origins; however, the concentrations of anthropogenic activities might be larger due to the fact that there are numerous industries, home activities, and agricultural activities along the Babon River.

Due to the unavailability of a regulated limit for metals in river sediment in Indonesia, this study employs international standards to facilitate the comparison of its findings. The levels of Cd in the sediment of the Babon River have been observed to be below the regulatory limits established by various countries, such as Australia (1.58 mg/kg) [42], the Netherlands (2 mg/kg), and the United States (9.6 mg/kg) [43]. The Babon River sediment's Cr contents were below Germany (100 mg/kg), Canada (111 mg/kg), and the Netherlands (250 mg/kg) regulations [44]. Pb in Babon River sediment is below the maximum allowable limit in Australia (50.8 mg/kg), the Netherlands (530 mg/kg), and the United States (218 mg/kg) [43].

The sediment of the Babon River contains significantly higher levels of Cd and Pb compared to the levels of these elements observed in rivers across Asia and Europe (Table 11). The Cr concentration in the Babon River sediment is relatively lower in comparison to the rivers in Southern China [45] and the rivers in Macedonia [47]. However, it appears that the Cr concentration exceeds that of the Thirumalairajan River in India [46] and Miliç Wetland, Türkiye [48]. The Fe contents in the Babon River sediment is comparatively lower than in several other rivers, such as Karnafully River, Bangladesh; Thirumalairajan River, India; Likova, Kumanova, and Pçinja Rivers, Macedonia; Guadalquivir River, Spain; and Miliç Wetland, Türkiye [40, 41, 46–48] (Table 11).

Our study found higher Cd and Pb concentrations in sediments with a dominant sand texture and higher Cr concentrations in sediments with a dominant clay texture. The texture of the sediment has an impact on its capacity to bind metals [19, 49]. Raj et al. [50] reported that sediments with a predominant clay texture in the Mahanadi estuary in India have high concentrations of Cd and Pb. Elevated concentrations of Fe are positively correlated with sediments composed primarily of sand texture in the Chennai littoral sediment, Tamil Nadu, India [51]. The presence of small mineral particles has a significant role in assessing the quality of water and sediment, especially in terms of metal adsorption [52, 53]. As a result of its greater surface area and capacity to exchange cations, fine particles in sediment frequently contain greater concentrations of trace metals than coarse particles [19]. In addition, fine particles such as clay in sediments are commonly bound to organic substances to generate organic-mineral complexes, which absorb metallic elements from water and subsequently settle to the sedimentary floor [54].

All sediment metals except Fe have an enrichment factor (EF) above 40, indicating extreme pollution. This suggests that the origin of enrichment for these metals in sediment is not derived from the crustal earth but rather from human sources. According to Szefer et al. [55], if the EF value exceeds 1.0, it may indicate that the metals are potentially mobilizing or diminishing if they fall in close proximity to the unity point of the crusted origin. Conversely, if the EF value exceeds 1.0, it might suggest that the chemical element originates from human activities. Furthermore, Nolting et al. [56] have suggested that EFs exceeding 10 may be attributed to a noncrusted source.

The assessment of the presence of metals in sediment can be performed by employing the Igeo method [57]. Igeo is a quantitative assessment of the pollution degree in a variety of soils and sediments. The Igeo scale consists of six classes that range from unpolluted to highly polluted [58]. Igeo results showed that sampling sites 1–7 had moderate Cd pollution, while sample sites 3, 4, 5, and 7 had moderate Pb pollution.

The analysis of concentration factors, contamination factors, enrichment factors, Igeo, and SPI data indicates that the sediment in the Babon River has been confirmed as being polluted with the elements Cd, Cr, and Pb. The evaluation of metal enrichment factors in sediments indicates a wide range of metal sources. However, it is likely that the concentrations of metals resulting from anthropogenic activities may be more pronounced in the Babon River region because of the presence of multiple industries, domestic activities, and agricultural practices. The Babon watershed has experienced a number of activities that potentially resulted in the production of metal effluent, hence causing the contamination of metals in the water and subsequent deposition in the Babon River. The upstream portion of the Babon watershed is mostly used for agricultural activities and rural residential communities, with a primary population of farmers. The central section of the river is primarily utilized for urban residential uses, alongside its agricultural utilization. Urban settlements and industrial areas are situated in the lower sections of the river. Sand and gravel extraction operations are present in the upper to central sections of the watershed [59].

The primary source of Cd content in the water and sediment of the Babon River may be linked to anthropogenic activities, since a number of industrial operations (such as metal, glass, plastic industries, tannery, and electroplating) have taken place along the river [60]. The presence of Cd in steel slabs and other metals, PVC, plastic, glass, and stamped materials is considered a potential source of Cd contamination in the aquatic environment [61]. In addition, the weathering and erosion of rocks transported by river flows contribute to the presence of Cd in the river [62]. According to Nriagu and Pacyna [63], it is believed that Cd has its origins in the process of air descending into bodies of water. According to GESAMP's research conducted in the Mediterranean Sea, the quantity of Cd intake from the air is nearly equivalent to the overall intake from rivers in the area [62]. Cadmium (Cd) and lead (Pb) are the metallic elements with the greatest contamination factor (CF) value in the Babon River. Consequently, they possess a tendency to accumulate in sediments. Furthermore, Cd is ranked second in terms of enrichment factor (EF), behind Cr. Hence, the sediment has experienced significant contamination with Cd. Cr is believed to originate from diverse anthropogenic sources such as ferrochrome production, electroplating for metal gilding, pigment synthesis, tanning, combustion of petroleum products, and waste incineration [64]. The origin of Cr is primarily attributed to the discharge of tanning wastewater [65] and the tanning practices carried out by local communities residing in the vicinity of the Babon River [60]. The principal factor contributing to the high concentration factor (Cf) of Cr in sediments is the presence of a favorable substrate. The correlation of sediment's Cr content and clay texture is significant, especially considering that the clay is the second most common type of texture after sand. Cr is the metallic element that experiences the most significant enrichment in sedimentary deposits [32]. However, the level of aggregation and enhancement that occurs remains restricted to the sediment concentrations found in the crustal earth, which prevents it from being classified as sediment that has been polluted by Cr. Pb is primarily sourced from the atmosphere through the combustion of petroleum products [66, 67]. It can enter the Babon River water through direct atmospheric deposition via rainfall, as well as indirectly through rainwater runoff, soil surface erosion, and other forms of land surface leaching by rainwater. These findings have been previously reported by WHO [64] and more recently by Haeruddin [8]. Lead (Pb) has the capacity to accumulate in sedimentary environments; however, its rate of enrichment is comparatively lower than that of chromium (Cr) and cadmium (Cd), resulting in moderate levels of sediment contamination. It is believed that the source of Fe metal is the weathered rocks of the crustal earth, in addition to anthropogenic sources, predominantly coal combustion residue from steam power plants located in close proximity to the Babon River estuary. The presence of Fe_2_O_3_, an iron oxidation substance, has been identified in fly ash [68]. Despite its potential for sediment accumulation, the degree of iron enrichment in sediments remains exceptionally low, thus excluding its classification as iron-contaminated sediment.

### 4.2. Assessment of Potential Ecological Hazards

The threshold effect level (TEL) is a statistical measure that is calculated from the geometric average of the 15^th^ percentile of contaminant concentrations that have been observed to elicit biological effects in various tests and the 50^th^ percentile of contaminant concentrations in tests where no biological effects were detected. The probable effect level (PEL) is a statistical measure that is generated from the geometric average of the 50^th^ percentile of contaminant concentrations that have been observed to have biological effects in various tests and the 85^th^ percentile of contaminant concentrations in tests where no biological effects were reported [69].

**Figure 4:**
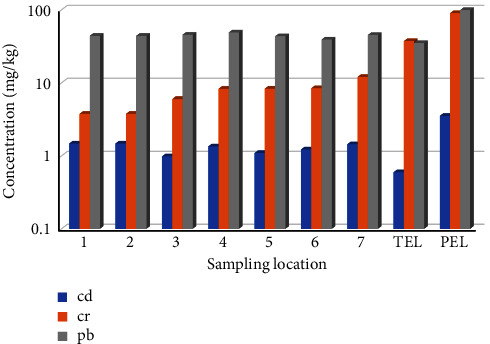
Comparison of Cd, Cr, and Pb concentrations in sediments with TEL and PEL.

The TEL denotes the threshold concentration at which unfavorable biological impacts are infrequently observed, while the PEL signifies the concentration level beyond which unfavorable impacts are anticipated to arise in a broader spectrum of living creatures. Based on the findings of Syakti et al. [70], the intermediate levels situated between TEL and PEL are linked with adverse biological effects, perhaps infrequently, occasionally, and frequently.  Figure 4 displays the TEL and PEL values for Cd, Cr, and Pb.

According to the TEL and PEL values, it can be considered that Cd and Pb metals exhibit the substantial toxicity with a rating (SQR) of 2, followed by Cr low toxicity with a rating (SQR) of 1. The concentration of Cd falls within the range of TEL-PEL at all sampling sites. The existence of Cd in the Babon River sediment has negative impacts on the biological well-being of the benthic biota, ranging from infrequent to frequent occurrences. The Cr content in the sediment at all of the sampling locations remains below the TEL and PEL regulatory limits. This means that the existence of Cr in the Babon River sediment does not have any adverse effects on the benthic biota. The levels of Pb in the river sediments are situated between the TEL and PEL. The potential biological consequences of Pb contamination in the Babon River sediment may be occasionally detected in the benthic environment. The potential for negative consequences resulting from the existence of metals in the Babon River sediment is primarily attributed to Cd and Pb. In the case where the metal concentration exceeds the natural concentration in sediment, detrimental consequences could occur for the aquatic biota, for example, a restriction of blood mussels and amphipods' ability to rebury themselves in sediments [21, 71].

## 5. Conclusion

The study's results indicated that the sediments of the Babon River consisted predominantly of sand and clay and that they had been contaminated with metals including Cd, Cr, and Pb. The pollution in the vicinity of the Babon watershed is thought to have been caused by human activities. These activities involve the transportation of water and sediment from the mainland or the direct introduction of contaminants into the river water via atmospheric and effluents of anthropogenic activity. Based on the examination of concentration factors, contamination factors, enrichment factors, Igeo, and SPI data, it has been substantiated that the sediment in the Babon River is polluted with Cd, Cr, and Pb. According to the TEL, PEL, and SQR values, it can be suggested that the presence of Cd and Pb has detrimental effects on the biological health of the benthic biota. The current study shows that the government must consider sediment metal contamination when developing river pollution legislation. By employing this method of assessment, the potential hazards presented by metals in river ecosystems are evaluated with greater precision, thus preventing increasingly severe adverse effects in subsequent periods.

## Figures and Tables

**Table 1:** Sampling sites and their surrounding areas.

Sampling site	Surrounding area
1	A mostly unpolluted water region borders the Penggaron forest. Riverbanks are sparsely inhabited and utilized for agriculture
2	Far from residential areas, it has less pollution. Plantation land surrounds the river
3	It is an area that is becoming contaminated due to its proximity to sparsely populated residential areas. The land on the banks of the river is utilized for traditional settlement and agricultural purposes
4	This area is becoming increasingly polluted due to its proximity to densely populated residential areas. However, the water quality remains high, making it a valuable source of raw material for drinking water
5	The area is heavily polluted due to its proximity to densely populated residential areas. The area surrounding the river is utilized for residential purposes and serves as a source of raw materials for the brick industry
6	The surrounding area is contaminated due to its traversal of densely populated residential areas. No industrial sector exists
7	It has been contaminated with industrial and domestic waste and is situated at the mouth of the Babon River

**Table 2:** Results (mg/L) obtained from seawater-certified reference materials' (NASS-7) analysis.

Metal	Certified value (mg/L)	Measured value (mg/L)	Recovery percentage (%)
Cd	0.0161 ± 0.0016	0.0148 ± 0.0015	92
Cr	0.107 ± 0.016	0.109 ± 0.016	102
Pb	0.0026 ± 0.0008	0.0025 ± 0.0008	96
Fe	0.351 ± 0.026	0.379 ± 0.028	108

Number of measurements (*n*) = 3.

**Table 3:** Results (mg/kg) obtained from sediment-certified reference materials' (PACS-3) analysis.

Metal	Certified value (mg/kg)	Measured value (mg/kg)	Recovery percentage (%)
Cd	2.23 ± 0.16	2.03 ± 0.14	91
Cr	90.6 ± 4.0	91.5 ± 3.9	101
Pb	188.0 ± 7.4	191.8 ± 8.1	102
Fe	41 060 ± 640	43526 ± 678	106

Number of measurements (*n*) = 3.

**Table 4:** Sediment quality ratings based on TEL and PEL [36].

SQR	Pb (mg/kg)	Cd (mg/kg)	Cr (mg/kg)
1	<35.000	<0.596	<37.300
2	35.000 < *x* ≤ 56.433	0.596 < *x* ≤ 1.574	37.300 < *x* ≤ 54.867
3	56.433 < *x* ≤ 77.867	1.574 < *x* ≤ 2.552	54.867 < *x* ≤ 72.433
4	77.867 < *x* ≤ 99.300	2.552 < *x* ≤ 3.530	72.433 < *x* ≤ 90.000
5	>99.300	>3.530	>90.000

**Table 5:** The concentrations of Cd, Cr, Pb, and Fe in river water and sediment at different sampling locations.

Sampling site	Cd		Cr		*P*b		Fe	
	Water^#^	Sediment^#^	Water^#^	Sediment^#^	Water^#^	Sediment^#^	Water^#^	Sediment^*∗*^
	(mg/L)	(mg/kg)	(mg/L)	(mg/kg)	(mg/L)	(mg/kg)	(mg/L)	(mg/kg)
1	0.0005 ± 0.000	1.483 ± 2.185	0.203 ± 0.351	3.766 ± 3.878	0.229 ± 0.142	44.208 ± 67.077	1.174 ± 0.053	976.368 ± 8.001
2	0.005 ± 0.004	0.602 ± 0.129	0.096 ± 0.165	5.201 ± 4.549	0.243 ± 0.091	47.774 ± 67.353	1.183 ± 0.042	906.870 ± 5.084
3	0.019 ± 0.025	0.987 ± 0.719	0.119 ± 0.103	6.018 ± 5.696	0.271 ± 0.060	45.774 ± 54.573	1.166 ± 0.066	967.284 ± 6.154
4	0.0255 ± 0.037	1.353 ± 0.970	0.279 ± 0.333	8.290 ± 7.243	0.280 ± 0.124	49.272 ± 65.954	1.153 ± 0.046	925.722 ± 26.651
5	0.036 ± 0.046	1.096 ± 1.177	0.229 ± 0.204	8.322 ± 8.438	0.419 ± 0.178	43.567 ± 49.261	1.185 ± 0.006	772.823 ± 32.924
6	0.051 ± 0.062	1.228 ± 1.391	0.259 ± 0.228	8.455 ± 9.223	0.323 ± 0.160	39.280 ± 48.788	1.619 ± 0.395	936.748 ± 7.532
7	0.061 ± 0.072	1.448 ± 1.303	0.334 ± 0.291	12.081 ± 12.383	0.311 ± 0.189	45.396 ± 49.041	1.174 ± 0.061	896.543 ± 5.898

^*∗*^Significant differences as determined by the Kruskal–Wallis test. ^#^There were no significant differences as determined by the Kruskal–Wallis test.

**Table 6:** Spearman correlation between sediment texture and metal concentration.

Metals	Texture					
	Sand		Silt		Clay	
	*r*	*P*	*r*	*P*	*r*	*P*
Cd	−0.091	0.694	0.512	0.018	−0.207	0.369
Cr	−0.247	0.280	0.022	0.924	0.263	0.249
Pb	0.829	0.050	0.388	0.082	−0.290	0.202
Fe	0.028	0.478	−0.095	0.681	−0.478	0.028

Note: *r* = Spearman correlation coefficient; *P* = significance level.

**Table 7:** Concentration factors (Cfs) of metals in the sediments' sampling sites.

Sampling location	Concentration factor			
	Cd	Cr	Pb	Fe
1	2965	2371	270	833
2	548	5635	297	767
3	448	35	210	830
4	788	27	322	804
5	363	24	170	652
6	329	21	215	603
7	367	25	297	765

**Table 8:** Contamination factors (CFs) of metals in the sediments' sampling sites.

Sampling location	Contamination factor			
	Cd	Cr	Pb	Fe
1	10.1	0.6	6.9	0.4
2	4.2	0.8	7.5	0.3
3	6.9	0.9	7.1	0.4
4	9.0	1.3	7.7	0.3
5	7.2	1.2	15.7	0.3
6	8.1	1.3	6.2	0.3
7	9.6	1.8	7.1	0.3

**Table 9:** Metal enrichment factors (EFs) in sediments at various sampling sites.

Sampling site	Enrichment factor (EF)			
	Cd	Cr	Pb	Fe
1	574	1453	214	1.10^−4^
2	250	2170	250	6.10^−5^
3	386	2356	224	9.10^−9^
4	556	3427	257	7.10^−5^
5	493	3425	197	7.10^−5^
6	493	3425	197	1.10^−4^
7	608	5066	238	2.10^−4^

**Table 10:** Geoaccumulation index (Igeo) of different metals at the sampling locations.

Sampling site	Igeo			
	Cd	Cr	Pb	Fe
1	1.9	−9.7	0.4	−5.9
2	2.0	−9.3	0.8	−6.0
3	2.5	−9.2	1.2	−5.9
4	2.9	−8.9	1.1	−5.9
5	2.3	−8.9	1.2	−6.2
6	2.4	−8.9	0.9	−5.9
7	2.9	−8.6	1.3	−6.0

**Table 11:** Concentrations of various metals (mg/kg) in the sediments of some rivers in Asia and Europe.

Metal in sediment (mg/kg)	Babon River	Rivers in South China [45]	Thirumalairajan River, India [46]	Karnafully River, Bangladesh [40]	Guadalquivir River, Spain [41]	Likova, Kumanova and Pçinja Rivers, Macedonia [47]	Miliç Wetland, Türkiye [48]
Cd	0.192–4.006	0.01–0.663	—	0.27–0.56	0.05–0.27	<1	0.19
Cr	0.0005–24.746	4.89–47.704	0.96–4.01	—	—	49.9–416	16.40
Pb	4.549–125.492	7.17–83.327	1.73–6.74	19.87–42.02	21.7–31	10.5–53.5	9.17
Fe	736.848–985.21	—	1736.3–3144	2723.38–3,798.76	15008–25633	31064–57020	10790

## Data Availability

The data used to support the findings of the study are available from the corresponding author upon request.
