# Corrigendum to “Spatial Distribution and Pollution Assessment of Metals in Sediments of the Babon River, Central Java, Indonesia”

**DOI:** 10.1155/sci5/9840693

**Published:** 2025-09-08

H. Haeruddin, A. Soegianto, F. Purwanti, A. Rahman, C. M. Payus, and H. Effendi, “Spatial Distribution and Pollution Assessment of Metals in Sediments of the Babon River, Central Java, Indonesia,” *Scientifica* 2024 (2024): 2065513, https://doi.org/10.1155/2024/2065513.

In the article, there is an error in the references. Specifically, reference 72 was mistakenly included by the authors and therefore should be omitted.

We apologize for this error.

